# Variation in coagulation factor activity levels cause discrepancies between activated partial thromboplastin time and anti-Xa activity for heparin monitoring: a retrospective observational study

**DOI:** 10.1186/s40560-023-00701-3

**Published:** 2023-11-16

**Authors:** Tomoyo Saito, Mineji Hayakawa, Osamu Kumano, Yoshinori Honma, Mone Murashita, Jun Kato, Syouki Fukui, Masaki Takahashi, Yuki Takahashi, Takumi Tsuchida, Asumi Mizugaki, Shuhei Takauji, Mariko Hayamizu, Tomonao Yoshida, Kenichi Katabami, Takeshi Wada, Kunihiko Maekawa

**Affiliations:** 1 Emergency and Critical Care Center, Hokkaido University Hospital, Sapporo, Japan; 2 Health and Medical Research Institute, National Institute of Advanced Industrial Science and Technology (AIST), Takamatsu, Japan

**Keywords:** Critical illness, Factor VIII, Factor Xa, Heparin, Partial thromboplastin time, Pseudo-heparin resistance

## Abstract

**Background:**

Unfractionated heparin (UFH) is primarily monitored using activated partial thromboplastin time (APTT). However, the recent introduction of anti-activated factor X (anti-Xa) activity testing has provided a direct evaluation of Xa inhibition by anticoagulants. This study aimed to investigate discrepancies between APTT and anti-Xa activity during UFH monitoring in critically ill patients and explore their underlying causes.

**Methods:**

This study analyzed 271 pairs of laboratory test results from blood samples of 99 critically ill patients receiving continuous intravenous UFH. Theoretical APTT values were calculated using fitted curve equations from spiked sample measurements with anti-Xa activity. Samples were categorized into three groups based on the measurement of the APTT/theoretical APTT ratio: the lower group (< 80%), the concordant group (80–120%), and the upper group (> 120%).

**Results:**

The overall concordance rate between APTT and anti-Xa activity was 45%, with a 55% discrepancy rate. The lower group frequently showed apparent heparin overdoses, while coagulation factor activities in the lower and upper groups were higher and lower, respectively, than those in the concordant group. Particularly, the lower group exhibited higher factor VIII activity levels than the upper and concordant groups.

**Conclusions:**

Discrepancies between APTT and anti-Xa activity were frequently observed, influenced by changes in coagulation factors activity levels. The lower and upper groups were classified as pseudo-heparin-resistant and coagulopathy types, respectively. Accurate monitoring of heparin in critically ill patients is crucial, especially in cases of pseudo-heparin resistance, where APTT values may wrongly indicate inadequate heparin dosing despite sufficient anti-Xa activity. Understanding these discrepancies is important for managing heparin therapy in critically ill patients.

*Trial registration*: Not applicable.

## Background

Anticoagulation therapy is frequently performed for critically ill patients. Although various anticoagulants have become available recently, unfractionated heparin (UFH) remains widely used and important in critically ill patients. UFH is primarily monitored using the activated partial thromboplastin time (APTT) [1]. Based on two small prospective studies conducted in the 1970s, a therapeutic APTT range of 1.5–2.5 times the control value has become widely accepted [2, 3]. However, APTT may be affected by various factors, including analytical variations in reagents and measuring instruments and biological factors, such as coagulation factors and acute-phase reactants [1, 4, 5]. It has also been reported that the sensitivity of APTT reagents to heparin varies according to differences in composition, and standardization has not been conducted [6, 7].

In contrast, anti-activated factor X (anti-Xa) activity is a pharmacokinetic test that directly evaluates Xa inhibition by anticoagulants. Thus, although the anti-Xa activity assay has not been standardized similarly to APTT tests, it should exhibit greater reproducibility and stability than the APTT assay, with analytical and biological variability [1, 8]. Several studies have shown that anti-Xa activity monitoring anticoagulation therapy with UFH is more useful than APTT [9–13]. In a meta-analysis of observational studies of patients on extracorporeal membrane oxygenation (ECMO), anti-Xa-based anticoagulation strategies were associated with fewer bleeding events and lower mortality rates without an increase in thrombotic events compared to time-based strategies like APTT [13]. However, in several countries, including Japan, anti-Xa activity is not commonly used to monitor UFH [14], and several guidelines recommend APTT measurements for UFH monitoring [15, 16]. Therefore, it is important to elucidate the relationship between APTT and anti-Xa activity.

Only a few studies have examined the relationship between anti-Xa activity and APTT [17–20]. The agreement between APTT and anti-Xa activity in the UFH therapeutic range was reported to be 54–65%, and discrepancies were frequently observed [17–20]. This phenomenon is easy to understand, as APTT, unlike anti-Xa activity, is a global coagulation test that can be influenced by the multiple coagulation factor activities and the anticoagulation effect of heparin [19]. However, to our knowledge, no study has examined the discrepancy between APTT and anti-Xa activity for anticoagulation monitoring in critical care settings. Thus, this study aimed to investigate the frequency of discrepancies between APTT and anti-Xa activity and determine the cause of this discrepancy in critical care settings.

## Methods

### Patients and blood samples

We retrospectively included critically ill patients who received continuous intravenous UFH and had their anti-Xa activity measured at the Emergency and Critical Care Center of Hokkaido University Hospital between March 1, 2019, and June 30, 2021. This study was approved by the Institutional Review Board of the Ethics Committee of the Hokkaido University Hospital (No. 021-0034, approved November 22, 2021). The need for informed consent was waived because of the study’s retrospective design. This study was conducted in accordance with the principles of the Declaration of Helsinki.

The target values and measurement timing of APTT and anti-Xa activity in each patient were determined by the attending physicians. Furthermore, heparin dosages were adjusted based on APTT and anti-Xa activity levels. Patients aged < 20 years were excluded or who received other anticoagulants (low-molecular heparin, other heparinoids, direct oral anticoagulants, warfarin, and nafamostat mesylate). Furthermore, data for blood samples without simultaneously measured APTT and anti-Xa activity data were excluded. Patient characteristics and laboratory test results were obtained from individual electronic medical records.

### Measurements

Anti-Xa activity and APTT were measured using an automated blood coagulation analyzer (CS-2500 [Sysmex Corporation, Kobe, Japan]) with BIOPHEN^™^ Heparin LRT (HYPHEN BioMed, Neuville-sur-Oise, France), which lacked antithrombin addition, and Thrombocheck APTT-SLA (Sysmex Corporation), respectively. Using the same automated blood coagulation analyzer, prothrombin time (PT), fibrinogen, fibrinogen/fibrin degradation products (FDP), and D-dimer levels were measured in clinical settings. Moreover, PT results were expressed as PT-international normalized ratios (PT-INR) for normalization. These measurement results were collected retrospectively.

After the measurements of anti-Xa activity, plasma samples were immediately collected and stored at – 80 ℃ until coagulation factor activity measurements. Coagulation factor activities in the stored plasma were measured using the automated blood coagulation analyzer with the PT or APTT methods.

### The relationship between anti-Xa activity and APTT in spiked samples

To investigate the relationship between anti-Xa activity and APTT in samples with normal coagulation factor activity, anti-Xa activity and APTT were simultaneously measured three times in five UFH-level samples (0.00, 0.36, 0.70, 1.00, and 1.33 IU/mL) using a BIOPHEN^™^ UFH Calibrator (HYPHEN BioMed, Neuville-sur-Oise, France). A third-degree polynomial equation was developed for curve fitting.

### Definition

To investigate the discrepancy between the measured APTT values and anti-Xa activity, the samples were categorized into three groups using the following methods: first, theoretical APTT values were calculated from a curve-fitting equation using measured anti-Xa activity results. Next, each sample’s APTT measurement/theoretical ratio was defined and calculated as the measured APTT/theoretical APTT (%). The samples were then categorized into the following three groups: the lower group (APTT measurement/theoretical ratio < 80%), the concordant group (APTT measurement/theoretical ratio 80–120%), and the upper group (APTT measurement/theoretical ratio > 120%).

### Data presentation and statistical analysis

Where appropriate, data were summarized using medians and interquartile ranges (25th–75th percentiles) or counts and percentages. The three groups were compared using Chi-square tests for categorical variables and Kruskal–Wallis tests for continuous variables. Post hoc analyses were conducted using the Mann–Whitney *U* test, with adjustments made for multiple comparisons through Bonferroni correction. Statistical analyses were performed using SPSS ver. 26 (IBM Japan, Tokyo, Japan), and the level of statistical significance was set at *p* < 0.05.

## Results

### Patients and blood samples

This study included 271 pairs of laboratory test results for plasma samples from 99 critically ill patients. Patient characteristics are presented in Table 1. The study population was typical of the profile of an emergency and critical care center cohort. The laboratory test results for all the samples are presented in Table 2.

**Table 1:** Characteristics of patients

	Patients number = 99
Age, year	69 (53–74)
Male, *n* (%)	68 (68.7)
Height, cm	165 (155–171)
Body weight, kg	65 (53–75)
APACHE II score	24 (18–32)
Primary disease for admission, n (%)	
Cardiac arrest	22 (22.2)
Cardiovascular disease	17 (17.2)
Sepsis	16 (16.2)
Burn	10 (10.1)
Trauma	7 (7.1)
Cerebral vascular disease	6 (6.1)
COVID-19	5 (5.1)
Pulmonary embolism	2 (2.0)
Other	14 (14.1)
Purpose for heparin administration, n (%)	
Prevention for DVT	34 (34.3)
ECMO	29 (29.3)
Atrial fibrillation	13 (13.1)
Treatment for DVT/PE	10 (10.1)
CRRT	7 (7.1)
Other	6 (6.1)
Heparin dosage (units/h)	600 (400–800)

*APACHE* Acute Physiology and Chronic Health Evaluation, *COVID-19* coronavirus disease 2019, *ECMO* extracorporeal membrane oxygenation, *DVT* deep venous thrombosis, *PE* pulmonary embolism, *CRRT* continuous renal replacement therapy

**Table 2:** Administrated heparin dosage and laboratory test results

	All samples *n* = 271	Lower group (APTT < Anti-Xa activity) *n* = 44	Concordant group (APTT = Anti-Xa activity) *n* = 121	Upper group (APTT > Anti-Xa activity) *n* = 106	*P* value
Heparin dosage (units/h)	600 (400–800)	800 (600–1000)^b^	624 (500–800)^b^	438 (400–600)^a,c^	< 0.001
White blood cell counts, 10^3^/mm^3^	10.4 (7.85–13.5)	10.0 (7.6–12.6)^b^	10.1 (7.7–13.2)	11.2 (8.2–14.5)^c^	0.331
Hemoglobin, g/dL	9.7 (8.9–11.3)	10.4 (9.3–12.5)	9.7 (8.6–11.6)	9.5 (8.8–10.3)	0.007
Platelet counts, 10^3^/mm^3^	149 (75–250)	210 (108–273)^b^	179 (102–308)^b^	79 (50–188)^a,c^	< 0.001
BUN, mg/dL	35 (25–49)	29 (20–42)	32 (25–50)	38 (27–51)	0.620
Creatinine, mg/dL	1.11 (0.73–2.19)	0.96 (0.67–1.57)	1.145 (0.73–2.41)	1.19 (0.75–2.19)	0.276
PT-INR	1.14 (1.08–1.26)	1.14 (1.08–1.27)	1.11 (1.05–1.21)^b^	1.19 (1.11–1.36)^a^	< 0.001
APTT, s	45.1 (37.5–58.8)	50.7 (38.1–60.3)^a,b^	37.8 (33.8–45.1)^b,c^	52.3 (45.1–71.0)^a,c^	< 0.001
Antithrombin, %	73 (63–88)	88 (75–96)^b^	76.5 (69–92)^b^	68 (54–76)^a,c^	< 0.001
Fibrinogen, mg/dL	419 (304–537)	439 (404–524)^b^	475 (385–579)^b^	347 (250–427)^a,c^	< 0.001
FDP, μg/mL	12.65 (7.4–27.7)	12.0 (8.2–19.6)	14.3 (6.2–34.4)	11.8 (7.9–21.5)	0.835
d-dimer, μg/mL	7.19 (3.9–15.035)	6.49 (3.55–10.25)	8.08 (3.96–19.52)	7.11 (3.94–12.92)	0.547
Coagulation factor activity, %					
Factor II	82 (67–95)	95 (84–105)^a,b^	88 (76–97)^b^	65 (51–78)^a,c^	< 0.001
Factor V	96 (75–121)	102 (91–130)^b^	105 (91–133)^b^	78 (60–100)^a,c^	< 0.001
Factor VII	69 (50–89)	72 (57–90)^b^	77 (61–95)^b^	57 (41–84)^a,c^	< 0.001
Factor VIII	144 (107–186)	178 (131–201)^b^	158 (123–201)^b^	126 (93–165)^a,c^	< 0.001
Factor VIII activity > 200%, *n* (%)	106 (39%)	16 (37%)	32 (26%)	58 (21%)	< 0.001
Factor IX	97 (76–112)	104 (94–116)^b^	108 (94–124)^#^	75 (58–96)^a,c^	< 0.001
Factor X	75 (62–88)	77 (68–90)^#^	80 (72–95)^#^	66 (55–77)^a,c^	< 0.001
Factor XI	72 (55–88)	86 (79–106)^#^	79 (67–96)	53 (40–64)^a,c^	< 0.001
Factor XII	40 (29–56)	52 (32–64)^#^	50 (35–65)^#^	32 (23–41)^a,c^	< 0.001
Anti-Xa activity (IU/mL)	0.23 (0.12–0.41)	0.63 (0.47–0.73)^a,b^	0.23 (0.11–0.34)^c^	0.18 (0.11–0.28)^c^	< 0.001

“APTT < Anti-Xa activity” indicates APTT measurement/theoretical ratio < 80%; “APTT = Anti-Xa activity” indicates APTT measurement/theoretical ratio 80–120%, “APTT > Anti-Xa activity” indicates APTT measurement/theoretical ratio > 120%. The theoretical APTT values were calculated from a curve-fitting equation using measured anti-Xa activity results. *BUN* blood urea nitrogen, *PT-INR* prothrombin time-international normalized ratio, *APTT* activated partial thromboplastin time, *FDP* fibrinogen/fibrin degradation products

^a^Statistical significance for the concordant group

^b^Statistical significance for the upper group

^c^Statistical significance for the lower group

### Relationship between anti-Xa activity and APTT

**Fig. 1:**
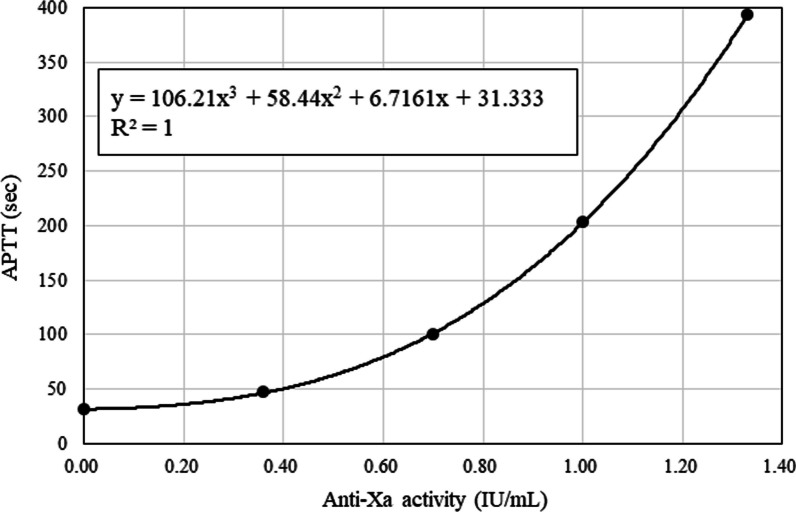
The relationship between anti-Xa activity and activated partial thromboplastin time in spiked samples. APTT, activated partial thromboplastin time

The relationship between anti-Xa activity and APTT in spiked samples is shown in Fig. 1. The equation for the curve fitting is as follows:

$${\text{y }} = \, 106.21{\text{x}}^{3} + \, 58.44{\text{x}}^{2} + \, 6.7161{\text{x }} + \, 31.333,{\text{ R}}^{2} \, = \, 1$$

$${\text{y}} = {\text{APTT }}\left( {\text{s}} \right){\text{ and x}} = {\text{anti}} - {\text{Xa activity }}\left( {\text{IU/mL}} \right).$$

**Fig. 2:**
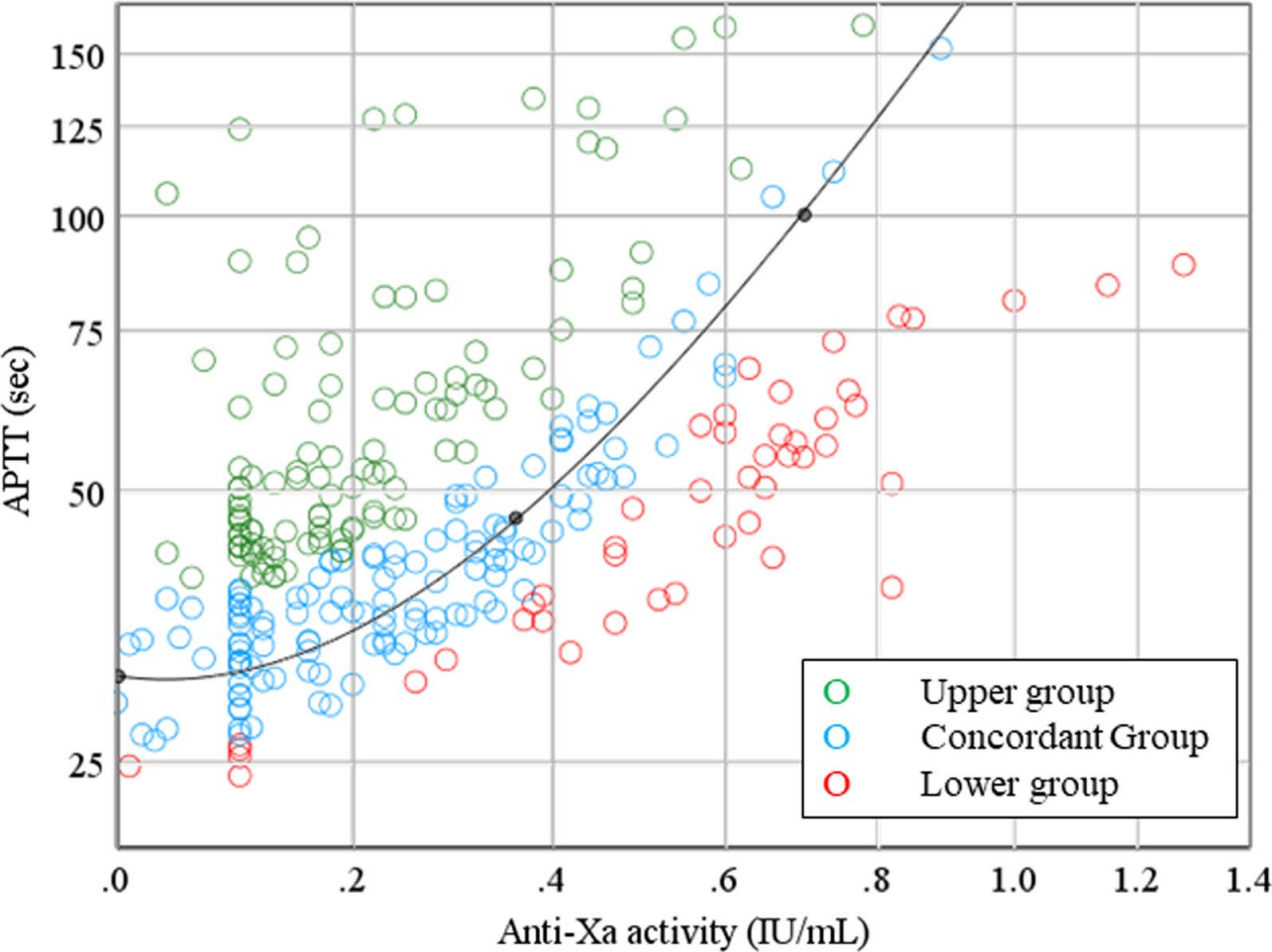
Scatter plot between anti-Xa activity and activated partial thromboplastin time. Green circle, upper group; blue circle, concordant group; red circle, lower group; black curve, the relationship between anti-Xa activity and APTT in normal plasma. APTT, activated partial thromboplastin time

**Fig. 3:**
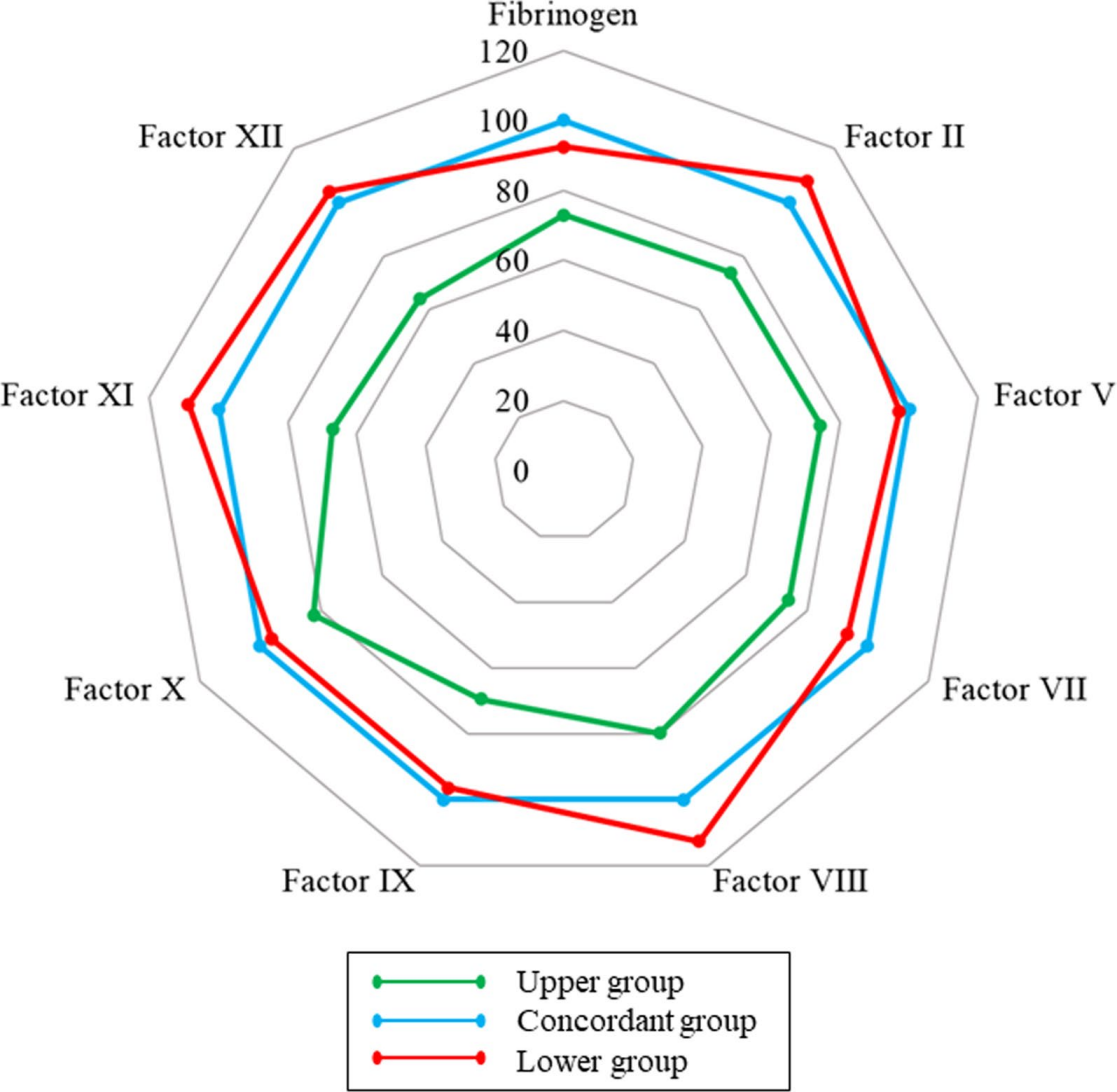
Radar chart for the comparison of median values in coagulation factor levels. The median value of each coagulation test in the concordant group was defined as 100%, and the median values in the other groups were expressed as values relative to the median in the concordant group. Green, upper group; blue, concordant group; red, lower group

A scatter plot between anti-Xa activity and APTT of all samples is presented with the classification of three groups: the lower group (red, *n* = 44), the concordant group (blue, *n* = 121), and the upper group (green, *n* = 106), as shown in Fig. 2. The proportion of patients in the concordant group was 45% (121/271). The percentages in the lower and upper groups were 16% (44/271) and 39% (106/271), respectively, indicating that 55% of the samples did not correspond to the theoretical values. In the lower group, apparent heparin overdoses were frequently observed (anti-Xa activity ≥ 0.7 was observed in 13 samples). A comparison of the laboratory test results among the three groups is presented in Table 2. Statistically significant differences were observed for all coagulation factors. The median value of coagulation factor activity in the concordant group was defined as 100%, and the relative median values were calculated for the upper and lower groups (Fig. 3). The activity levels of all coagulation factors were lower in the upper group than in the concordant group. In contrast, the activity levels of factors II, VIII, and XI in the lower group were slightly higher than those in the concordant group. In particular, the percentage of samples with factor VIII activity levels > 200% was 37%, much higher than that of the other two groups.

## Discussion

This study evaluated the relationship between APTT and anti-Xa activity, and the cause of the discrepancy was investigated in samples from critically ill patients. The percentage of samples in the concordant groups was 45%, indicating that more than 50% of the samples did not correspond to the measured APTT level to the theoretical APTT levels calculated by anti-Xa activity. The lower group showed higher coagulation factor activity levels, especially factor VIII, and were considered to have pseudo-heparin resistance. In the pseudo-heparin resistance group, apparent heparin overdoses were frequently observed.

In the acute phase of critical illness, coagulopathy induced by bleeding, hepatic failure, and disseminated intravascular coagulation are frequently observed [21–24]. In these disorders, coagulation and fibrinolytic reactions occur, and coagulation factor activity levels decrease due to their consumption during coagulation activation. APTT was prolonged in these patient samples. Moreover, this study observed coagulation and fibrinolysis activation because an increase in FDP and d-dimer levels and APTT prolongation were confirmed (Table 2). Importantly, a prolonged APTT relative to anti-Xa activity is frequently observed. In addition, severe antithrombin deficiency induces heparin resistance, which is detected as a low anticoagulation effect relative to the amount of heparin administration [25–27]. Notably, decreased antithrombin activity levels are frequently observed in the acute phase in critically ill patients [23, 24]. Although moderate decreases in antithrombin activity levels were frequently observed in the upper group, there was no severe deficiency in antithrombin samples in this study (Table 2). Furthermore, large amounts of heparin are not administered to patients with severe deficiencies due to severe coagulopathy. In the upper group, the discrepancy is due to lower coagulation factor activity levels arising from the consumption of these factors; this is classified as a type of coagulopathy.

Fibrinogen and coagulation factor VIII are acute-phase proteins that increase gradually in response to inflammation [28]. Fibrinogen synthesized in the liver is mainly upregulated by interleukin 6 released after inflammation [28]. The synthesis of coagulation factor VIII is observed not only in hepatocytes, but also in the kidneys, endothelial cells, and lymphatic tissue [29, 30] and is upregulated by inflammatory stimulation [31]. In the present study, both fibrinogen and factor VIII levels were markedly elevated, and these elevations, derived from the inflammatory stimulation of critically ill patients, would affect the discrepancy between APTT and anti-Xa activity in the lower group. In the lower group, when anti-Xa activity reached sufficient levels for anticoagulation, APTT was not prolonged. Thus, this group was classified as pseudo-heparin resistant. If UFH is monitored only using APTT in patients with pseudo-heparin resistance, the over-administration of UFH will be induced.

In the lower group (pseudo-heparin resistance type), the discrepancy between APTT and anti-Xa activity might be affected by UFH clearance in vivo. UFH is heterogeneous in terms of molecular weight, pharmacokinetic properties, and anticoagulation activities [32]. The range of UFH molecular weight spans from 3000 to 30,000 Da, and in vivo clearance of UFH is affected by molecular weight [32]. The clearance of high-molecular-weight heparin is greater than that of low-molecular-weight heparin. Furthermore, the clearance of low-molecular-weight heparin is more sensitive to renal function than that of high-molecular-weight heparin [33]. Moreover, the anticoagulation activity of low-molecular-weight heparin is not reflected in APTT [32]. Therefore, in the lower group (pseudo-heparin resistance type), the increased concentration of low-molecular-weight heparin due to differences in in vivo clearance by molecular weight may have contributed to the discrepancy between APTT and anti-Xa activity.

APTT is used to screen for the intrinsic and common pathways involved in the coagulation cascade. However, the effect on APTT prolongation differs for each coagulation factor [34–36]. Lawrie et al. investigated the sensitivity of the APTT reagent to each factor based on the Clinical and Laboratory Standard Institute guidelines and reported that coagulation factors that show a clotting time in the upper limit of the reference interval were factors VIII, IX, XI, and XII at levels of 31%, 31%, 52%, and 14%, respectively [37]. Another study indicated that APTT prolongation was observed for factor V activity levels < 45%, factors II and XI activity levels < 40%, and factors I, V, VII, VIII, IX, and XII activity levels < 25% [36]. This indicated that a difference in the sensitivity for each coagulation factor, including extrinsic and common pathways similar to the intrinsic pathway, existed in APTT reagents. Therefore, APTT prolongation would differ even if the activity levels were the same among the coagulation factors [36, 37]. This study confirmed that coagulation factor activity levels differed among the three groups, and a large difference was observed between the upper and lower/concordant groups. In the lower group, it was considered that factors II, VIII, and XI could significantly affect APTT because the levels of these three factors in the group were higher than those in the concordant group.

This study had some limitations. First, this study had a small sample size and was conducted at a single center. Second, clinical outcomes such as thrombotic events and bleeding complications were not evaluated. Third, the coagulation factor VIII activity level in many samples exceeded the upper measurement limit and could not be measured accurately. Unfortunately, due to insufficient sample volume, additional diluted measurements were not possible. Fourth, detailed coagulation characteristics of patients before heparin administration were not demonstrated because of a lack of sample collection for the study. The detailed association between coagulopathy and APTT prolongation in critically ill patients without heparin is unclear.

## Conclusions

In critical care settings, discrepancies between APTT and anti-Xa activity are frequently observed. The causes of discrepancy are coagulopathy in the upper group and pseudo-heparin resistance in the lower group. Coagulopathy type discrepancy was more frequent than pseudo-heparin resistance type discrepancy. Therefore, it is important to understand the differences between APTT and anti-Xa activity during heparin monitoring.

## Data Availability

The data supporting the findings of this study are available from the corresponding author, M Hayakawa, upon reasonable request.

## Abbreviations

Anti-Xa activity: Anti-activated factor X activity
APTT: Activated partial thromboplastin time
ECMO: Extracorporeal membrane oxygenation
FDP: Fibrinogen/fibrin degradation products
PT-INR: Prothrombin time-international normalized ratio
